# Inferring Association Between Alcohol Addiction and Defendant's Emotion Based on Sound at Court

**DOI:** 10.3389/fpsyg.2021.669780

**Published:** 2021-05-28

**Authors:** Yun Song, Zhongyu Wei

**Affiliations:** ^1^Law School, Heilongjiang University, Harbin, China; ^2^School of Data Science, Fudan University, Shanghai, China

**Keywords:** alcohol addiction, emotion prediction, AI in law, defendant's emotion, deep learning

## Abstract

Alcohol addiction can lead to health and social problems. It can also affect people's emotions. Emotion plays a key role in human communications. It is important to recognize the people's emotions at the court and infer the association between the people's emotions and the alcohol addiction. However, it is challenging to recognize people's emotions efficiently in the courtroom. Furthermore, to the best of our knowledge, no existing work is about the association between alcohol addiction and people's emotions at court. In this paper, we propose a deep learning framework for predicting people's emotions based on sound perception, named ResCNN-SER. The proposed model combines several neural network-based components to extract the features of the speech signals and predict the emotions. The evaluation shows that the proposed model performs better than existing methods. By applying ResCNN-SER for emotion recognition based on people's voices at court, we infer the association between alcohol addiction and the defendant's emotion at court. Based on the sound source data from 54 trial records, we found that the defendants with alcohol addiction tend to get angry or fearful more easily at court comparing with defendants without alcohol addiction.

## 1. Introduction

Emotion perception plays a key role in human communications and is hence of interest to a range of disciplines, including psychology, psychiatry, and social neuroscience. Particularly, emotions may influence thinking patterns, decision-making, and talking. At the court, the talking and thinking pattern of defendants are critical for judges to make a decision. Therefore, it is important to study the role of the defendants' emotions at the court and which factors may affect the defendants' emotions. Nowadays, alcohol addiction is one of the most common substance use disorders. Alcohol addiction may impact several aspects of people's daily life, including people's emotions. The existing study shows that emotions are likely to affect court judgment when a particular information processing style is active (Nunez et al., 2016). Studying the relation between alcohol addiction and the defendants' emotions at court can help to answer several key questions. Does alcohol addiction affect the defendants' emotions? How can we avoid the bias resulting from the emotions of people, such as lawyers, judges, defendants, plaintiffs, and so on?

The existing study has shown that emotions are associated with alcohol addiction. However, few studies can be identified regarding the association between alcohol addiction and the defendant's emotion at court. Recognizing people's emotions at court precisely is a prerequisite while studying the association between the alcohol addiction and the defendant's emotion at the court. There are several ways to recognize people's emotions, including face-based, touch-based, vision-based, and questionnaire-based (Schirmer and Adolphs, 2017). However, most of them are not available in the specific context of the court. At the court, there are some specific rules. Speech is one of the signals we can capture easily at the court. Therefore, the speech-based method is one of the most appropriate methods to recognize emotions.

In this paper, we propose a deep learning framework for predicting people's emotions based on speech (voice), which could be easily applied to recognize the people's emotions at court and used to study the association between alcohol addiction and the defendant's emotion at court.

Currently, emotion recognition based on speech has been an attractive research topic in artificial intelligence due to its importance in lots of research areas, such as psychology, psychiatry, and social neuroscience. Several methods have been proposed to recognize emotion based on speech. The task of emotion recognition based on speech usually contains two main steps (Koolagudi and Rao, 2012). The first step is feature extraction. The second step is emotion classification. Feature extraction is a key step to fill the affective gap between digital signals and subjective emotions. Several types of hand-designed features have been proposed for speech-based recognition, including source-based excitation features, prosodic features, vocal traction factors, and other hybrid features. For the classification step, both linear and non-linear machine learning algorithms are widely used to distinguish the underlying emotion categories. So far, the classifiers used for emotion recognition from speech include Bayesian networks (BN), the maximum likelihood principle (MLP), support vector machine (SVM), Gaussian Mixture Model (GMM), Hidden Markov Model (HMM), and so on (Khalil et al., 2019).

Feature extraction is very important in speech emotion recognition (SER). Although researchers have made a lot of effort on hand-designed features, the hand-designed features are usually low-level features. These features may not be enough to recognize different emotions. It is urgent to extract the features automatically that can represent high-level features of speech. Inspired by the success of deep learning on image analysis and natural language processing, deep learning methods provide a possible solution for automatical feature extraction to address this problem. Comparing with the traditional methods, deep learning-based methods have several strengths, including the ability of handling complex structures, the discovery of hidden features, and removing unrelated noise.

Deep Neural Network (DNN) is a deep learning method used for feature extraction of speech signals at the early stage. DNN contains several layers of neural networks. For example, Han et al. (2014) use a DNN-based model to extract the feature of the speech signals and recognize the emotions. Convolutional neural network (CNN) is one of the widely used deep learning methods, which are commonly used for feature extraction and pattern recognition (Dong et al., 2014). Recently, CNN has been successfully applied to extracting the feature of speech signals automatically. Abdel-Hamid et al. use a one-layer CNN for feature extraction, which achieves good performance. Huang et al. (2014) combine CNN with sparse auto-encoder to extract high-level features for SER. However, one drawback is that CNN cannot capture the relations between different time points. To address this problem, Trigeorgis et al. (2016) propose an end-to-end model based on a two-layer CNN and long short-term memory (LSTM) to extract speech features and recognize emotions. LSTM is a recurrent neural network (RNN) architecture designed to process time-series data. The basic idea is that LSTM can remember values over arbitrary intervals. Huang et al. (2018) utilize the LSTM to capture values over arbitrary intervals and extract features. Those features, then, are fed into SVMs to recognize emotions.

Although one-layer, two-layer CNN or LSTM-based models are widely used for speech-based emotion recognition, these models are shallow ones. Comparing with the shallow models, models with deep multi-level convolutional and pooling layers can extract features more exactly. The reason is that deep structure can capture the hierarchical structure of information contained in the speech signals. To address this problem, Lim et al. (2016) propose a deep hierarchical feature extraction model by combining a CNN with LSTM. By combining principal component analysis and deep CNN, Zheng et al. integrate the linear and non-linear model to capture the features of speech signals (Barros et al., 2015). Tzirakis et. al propose a deep model to recognize various emotions using a CNN and ResNet of 50 layers (Badshah et al., 2017). Tang et al. (2018) achieve good performance by combining a CNN and a RNN with ResNet. So far, a lot of methods are proposed for recognizing emotions based on speech signals only. However, these existing models are not efficient enough to be applied in recognizing the emotions of people at court precisely. Generally, the drawbacks of existing methods are as follows: (1) although several methods integrate different types of models including CNN, LSTM, and so on, they are not designed well and cannot effectively integrate different types of models; (2) the existing methods fail to consider which part of the speech signal is more important for the emotion recognition, which may lead to the inaccurate result. Therefore, a proper method is needed to integrate multiple types of neural network-based models effectively and consider the importance of different parts of the speech signal.

In this study, we propose a DNN-based framework, named ResCNN-SER, which includes convolutional networks with residual structure and LSTM. To model the importance of different parts of the speech signal, an attention-based model is also integrated into the proposed model. By using ResCNN-SER, we can predict the defendant's emotions at court and analyze the association between the alcohol addiction and the defendant's emotion. To the best of our knowledge, there is no dataset generated for this purpose. Therefore, we collect 54 sound records of the trial at court. Based on the dataset, we analyze the association between alcohol addiction and people's emotions at court using the proposed method ResCNN-SER. The major contributions are listed as follows:

- We propose a deep learning framework for speech-based emotion recognition, which can be used to predict people's emotions at court. The evaluation results show that the proposed model outperforms the state-of-art method for speech-based emotion recognition.
- Using the proposed method, we analyze the association between alcohol addiction and people's emotions at court. The results show that people who have alcohol addiction problem are more likely to be angry and fearful at court.

## 2. Method

Our work mainly includes two parts: (1) proposing a novel method to recognize emotions based on speech signals; (2) analyzing the association between alcohol addiction and people's emotions at court. In the first part, the proposed model uses the CNN, LSTM, and attention mechanism to capture the raw features, and consider the importance of different parts of speech signals, respectively. In the second part, we generate and manually annotate a dataset including 54 sound records of trials at court. Based on the proposed method and generated dataset, we can analyze the association between alcohol addiction and emotions at court.

### 2.1. A Novel Method to Recognize Emotions Based on Speech Signal

**Figure 1:**
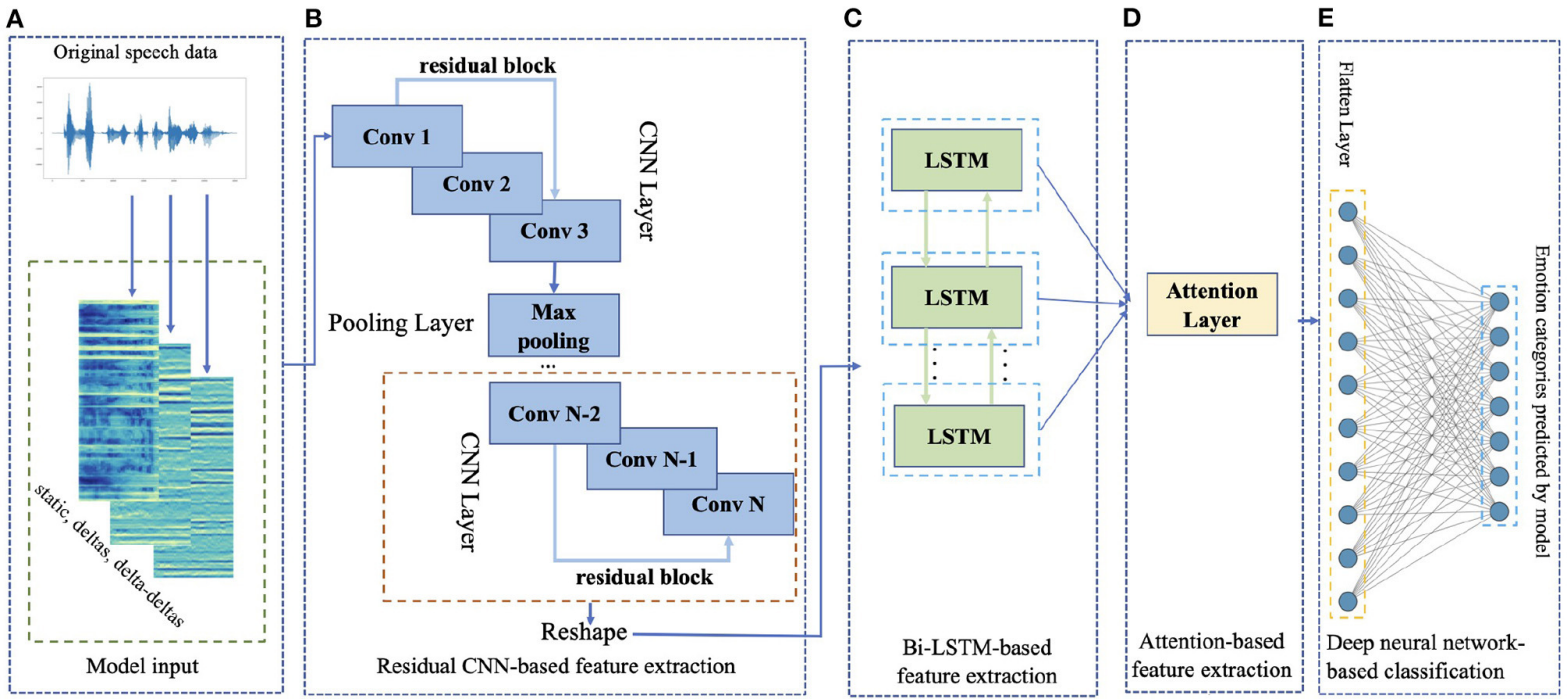
The framework of ResCNN-SER. An illustrative example is shown to describe the ResCNN-SER model. **(A)** We first compute the log-Mels spectrogram (static, deltas, and delta-deltas). The log-Mels spectrogram feature can be considered as an image. **(B)** Taken the log-Mels spectrogram as the input data, we use a convolutional neural network (CNN) model to extract the features. **(C)** A bidirectional long short-term memory (Bi-LSTM) model is applied to extract the features by considering the information at different time points. **(D)** We apply an attention model to enhance the feature representation. **(E)** A fully connected neural networks layer is used to predict the speech emotion categories.

We propose a novel algorithm called ResCNN-SER to recognize emotions based on the speech signal. The framework of ResCNN-SER is shown in Figure 1. The proposed framework mainly contains five components. First, given the speech signal sequence data (Figure 1A), we calculate the log-Mels spectrogram (static, deltas, and delta-deltas) as the representation of the input signal. Second, we apply the CNN with residual block to extract the feature of log-Mels with deltas and delta-deltas data (Figure 1B). Third, in order to consider the sequence information at different time points, we use a bidirectional long short-term memory (Bi-LSTM) model to extract the features (Figure 1C). Fourth, in order to enhance the feature representation, we apply an attention model (Figure 1D). Finally, we use a fully connected layer to predict the speech emotion categories (Figure 1E).

#### 2.1.1. Log-Mels Spectrogram Feature Calculation

The quality of features extracted from speech data is crucial for the performance of SER. Many existing studies have shown that the performance of SER task can be improved by converting the speech signal sequence data into log-Mels spectrogram data (Zhang et al., 2017; Chen et al., 2018; Dai et al., 2019; Zhao et al., 2019; Zhou et al., 2019; Chen and Zhao, 2020; Zayene et al., 2020). In this way, it can reduce the difficulty of SER caused by different speaker styles and intonations (Chen et al., 2018).

In this paper, inspired by previous work (Chen et al., 2018), we calculate the log-Mels spectrogram of speech signal sequence data. In details, given a speech signal sequence data, we use the z-score standardization method to standardize the speech signals from different speakers. The speech signals are split into short frames. We set the size of hamming windows as 25 *ms* and the step length of shift as 10 *ms*. For each short frame, the discrete Fourier transform (DFT) is applied to calculate the power spectrum. The power spectrum of frame *i* is fed to Mel-filter bank to obtain *p*_*i*_. Then, the logarithm operation is applied to *p*_*i*_ as follows:

(1) $$\begin{array}{r} m_{i}=\log\left(p_{i}\right) \end{array}$$

Based on this, we calculate the deltas features of the static log-Mels *m*_*i*_. Given *m*_*i*_, we use the following formula to calculate deltas features $m_{i}^{d}$ as follows:

(2) $$\begin{array}{r} m_{i}^{d}=\frac{\sum_{n=1}^{N}n\left(m_{i+n}-m_{i-n}\right)}{2\sum_{n=1}^{N}n^{2}} \end{array}$$

Where *i*, *d* represent the number of frames and deltas, respectively. Inspired by Keren and Schuller (2016), the *N* is set as 2. Finally, we calculate deltas–deltas features $m_{i}^{dd}$ based on the $m_{i}^{d}$. The formula is shown as follows:

(3) $$\begin{array}{r} m_{i}^{dd}=\frac{\sum_{n=1}^{N}n\left(m_{i+n}^{d}-m_{i+n}^{d}\right)}{2\sum_{n=1}^{N}n^{2}} \end{array}$$

After calculating the *m*_*i*_, $m_{i}^{d}$ and $m_{i}^{dd}$, all these features are merged together. A 3-D feature representation *M* ∈ ℝ^t*f*c^ of the given speech signal is generated. *t* represents the length of time frame. *f* represents the number of Mel-filter bank. *c* represents the number of feature channels. In this case, the number of feature channels is three, which are static, deltas, and delta-deltas, respectively.

#### 2.1.2. Residual Block CNN-Based Feature Extraction

CNNs (Lavin and Gray, 2016) have made great success in several fields, such as computer vision, natural language processing, and bioinformatics. Lots of DNN-based models with multiple convolutional layers have been constructed. However, there might be a loss of feature information during the information passing process from layer to layer if the model has too many layers. To overcome this problem, several approaches have been proposed in computer vision, natural language processing area. For example, a long short-term memory network (LSTM) (Hochreiter and Schmidhuber, 1997; Gers et al., 1999) model is designed to prevent the information loss in the traditional RNN (Pearlmutter, 1989). A residual structure (ResNet) is designed to prevent the loss of information in the deep CNN (He et al., 2016). In this paper, inspired by the ResNet model, we adopt a similar residual block structure to prevent the loss of feature information when extracting the speech signal features.

In the proposed ResCNN-SER framework, we use the CNN with residual block model to extract the feature of the speech signal. Given a 3-D feature representation *M* ∈ ℝ^t*f*c^, it can be considered as an image input, which has *c* channels. The shape of the image is t*f. In the proposed CNN model with residual blocks, we first use 128 kernels with kernel size 1*1 to improve the channel size. Then we use two convolutional layers to extract the feature. Each convolutional layer has 128 kernels with kernel size 3*3. A residual block is used to avoid information loss. In this block, we add the output of the first convolutional layer and the third convolutional layer. After that, we use a max-pooling layer for dimension reduction, which size is 2^*^4 and stride is 2^*^4. Three residual blocks are included in the model.

#### 2.1.3. Bi-LSTM-Based Feature Extraction

LSTM (Hochreiter and Schmidhuber, 1997; Gers et al., 1999) is one type of RNN-based model, which has been used in several areas. In natural language processing (NLP), LSTM is used to capture the semantic information between different words (Wang et al., 2016). In tasks for processing time-series data, LSTM can extract the information between data from different time points (Gers et al., 2002; Karim et al., 2019). In this paper, we use a bidirectional LSTM (BiLSTM) model (Graves and Schmidhuber, 2005; Huang et al., 2015; Zhou et al., 2016) to extract the features. BiLSTM includes two components: the forward LSTM and the backward LSTM. This model can capture both previous information and future information.

LSTM is a gated neural network, which includes the forget gate, input gate, and output gate. At time point *t*, the output of the hidden layer may be related to the previous information (before t) or future information (after t). Forget gate and input gate are designed for remaining previous information and current information, respectively. By combining the results of the forget gate and the input gate, the output gate generate a hidden layer as output.

Mathematically, forget gate is implemented as follows:

(4) $$\begin{array}{r} f_{t}=\sigma \left(W_{cf}c_{t-1}+W_{hf}h_{t-1}+W_{xf}x_{t}+b_{f}\right) \end{array}$$

Input gate is implemented as follows:

(5) $$\begin{array}{r} i_{t}=\sigma \left(W_{ci}c_{t-1}+W_{hi}h_{t-1}+W_{xi}x_{t}+b_{i}\right) \end{array}$$

(6) $$\begin{array}{r} c_{t}=f_{t}c_{t-1}+i_{t}\tanh\left(W_{hc}h_{t-1}+W_{xc}x_{t}+b_{c}\right) \end{array}$$

Output gate is implemented as follows:

(7) $$\begin{array}{r} o_{t}=\sigma \left(W_{co}c_{t}+W_{ho}h_{t-1}+W_{xo}x_{t}+b_{o}\right) \end{array}$$

(8) $$\begin{array}{r} h_{t}=o_{t}\tanh\left(c_{t}\right) \end{array}$$

where σ represents the sigmoid activation function, *x*_*t*_ represents the inputs at time *t*. *c*_*t*_, and *c*_*t*−1_ represent the state at time *t* and *t* − 1, respectively. *h*_*t*_ and *h*_*t*−1_ are hidden layer outputs at time *t* and *t* − 1. *W* and *b* are the trainable parameters, respectively. *f*_*t*_, *i*_*t*_, *o*_*t*_ are the outputs of the forget gate, input gate, and output gate, respectively. Given an output of the LSTM layer, we use a Bi-LSTM model to extract the features. Both the forward LSTM layer and the backward LSTM layer have 128 hidden units, respectively. Therefore, the dimension of the output feature for Bi-LSTM is 256.

#### 2.1.4. Attention-Based Method to Enhance the Feature Representation

In this step, we use the attention mechanism to enhance the feature representation. The attention method has made great success in many fields, such as computer version (Woo et al., 2018; Bello et al., 2019) and natural language processing (Yin et al., 2016; Vaswani et al., 2017). Inspired by these methods, we adopt the attention method in our model.

Given a Bi-LSTM layer output $h_{t}=\left[\overset{⃖}{h_{t}},\vec{h_{t}}\right]$, where *h*_*t*_ represents the feature representation at time step *t* in two directions. We calculate the importance score α_*t*_ of *h*_*t*_. In detail, we use the softmax function to calculate the α_*t*_ as follows:

(9) $$\begin{array}{r} \alpha_{t}=\frac{e^{\sigma \left(W_{1}*h_{t}+b\right)}}{\sum_{i=1}^{T}e^{\sigma \left(W_{1}*h_{i}+b\right)}} \end{array}$$

where σ is the sigmoid function, σ(*x*) = 1/(1+*e*^−*x*^), *W*_1_ and *b* are trainable parameters. After calculating the importance score α_*t*_ of *h*_*t*_, we can compute the representation *r* as follows:

(10) $$\begin{array}{r} r=\overset{T}{\underset{t=1}{\sum }}\alpha_{t}h_{t} \end{array}$$

#### 2.1.5. Fully Connected Neural Network-Based Speech Emotion Recognition

After the attention layer, we use the fully connected neural network to predict the speech emotions. Given a feature representation resulted from the attention layer, we use a two-layer neural network to predict the emotions based on speech signals. At the last layer, we can get the predicted label *p*_*ic*_ by the softmax function. Then we use the cross-entropy loss (Zhang and Sabuncu, 2018) to train the ResCNN-SER model. The loss function can be calculated as follows:

(11) $$\begin{array}{r} L=-\frac{1}{N}\overset{N}{\underset{i=1}{\sum }}\overset{c}{\underset{c=1}{\sum }}y_{ic}\log\left(p_{ic}\right) \end{array}$$

where *c* is the number of categories, *y*_*ic*_ is the true label, *p*_*ic*_ is the predicted label, and *N* is the number of samples.

### 2.2. Dataset for Analyzing Association Between Alcohol Addiction and Emotion at Court

We study the association between alcohol addiction and emotions at court following three steps. First, a dataset designed for the research purpose is collected. Second, we manually annotate the dataset. Third, the ResCNN-SER is trained on the annotated dataset and applied to the rest of the dataset to analyze the association between alcohol addiction and emotions at court.

#### 2.2.1. Sound at Court Dataset Generation

Since there is no existing dataset designed for studying the association between alcohol addiction and emotions at court, we generate a dataset including the sounds of 100 trial records at court from Datong People's Court, Daqing, Heilongjiang, China. These trial records are publicly available at http://tingshen.court.gov.cn. The lengths of these trial records range from 10 to 60 min. There are three types of speakers in the dataset, including judges, plaintiffs, and defendants. Two pre-process steps are performed. First, we remove the long blank stops between two sentences. The length of stops between two sentences is set as 1 s. Then, for each record, the sentences are separated into three groups for judges, plaintiffs, and defendants, respectively.

#### 2.2.2. Dataset Annotation

To analyze the association between alcohol addiction and emotions at the court, it is necessary to annotate people with and without alcohol addiction problems. Therefore, we first go through all 100 trial records manually and annotate people with alcohol addiction. In these trial records, 27 records contained people with an alcohol addiction problem. It is expected that all people having an alcohol addiction problem are defendants. Because it is time consuming to recognize people's emotions by experts manually, we use the proposed method, named ResCNN-SER, to recognize people's emotions automatically based on the speeches at court. To train the ResCNN-SER model, we labeled speeches in ten trial records as the training data. To simplify the problem, if a person expressed one type of emotion *M* except neutral, we labeled the person as the type of emotion *M*. For each trial record, three experts are invited to annotate the speeches. For each speech, if more than two experts label the speech as the same emotion, the speech is labeled as that emotion. After using the labeled data to train the ResCNN-SER model, we can use the ResCNN-SER model to analyze the left dataset automatically.

Our goal is to analyze the association between alcohol addiction and emotion at court. Corresponding to 27 records contained people with alcohol addiction, 27 records are selected from the other 73 records. In detail, in both alcohol addiction and non-alcohol addiction data sets, people's emotions are recognized based on ResCNN-SER. Based on the generated datasets, we can analyze the association between alcohol addiction and emotions at court.

## 3. Results and Discussion

### 3.1. Evaluation of ResCNN-SER

#### 3.1.1. Data Preparation

There is no publicly available data set for the evaluation of emotion recognition based on sound perception at the court. Therefore, for a fair comparison, we use a publicly available dataset that is generated for emotion recognition based on sound perception. To evaluate the performance of ResCNN-SER, we test our model on the Emo-DB dataset, which is derived from Berlin Emotional Database (Burkhardt et al., 2005). The dataset is widely used for evaluating SER algorithms in previous studies. The emo-DB dataset has 535 utterances. These utterances were spoken by ten professional actors. Every utterance has one label. These labels are sadness, disgust, boredom, neutral, fear, joy, or anger. In other words, the dataset includes seven emotions. In the experiment, to model these labels in the proposed method, we use the one-hot to encode these labels. More details of the Emo-DB dataset can be found on the website (http://emodb.bilderbar.info/index-1280.html).

#### 3.1.2. Evaluation Metrics

**Figure 2:**
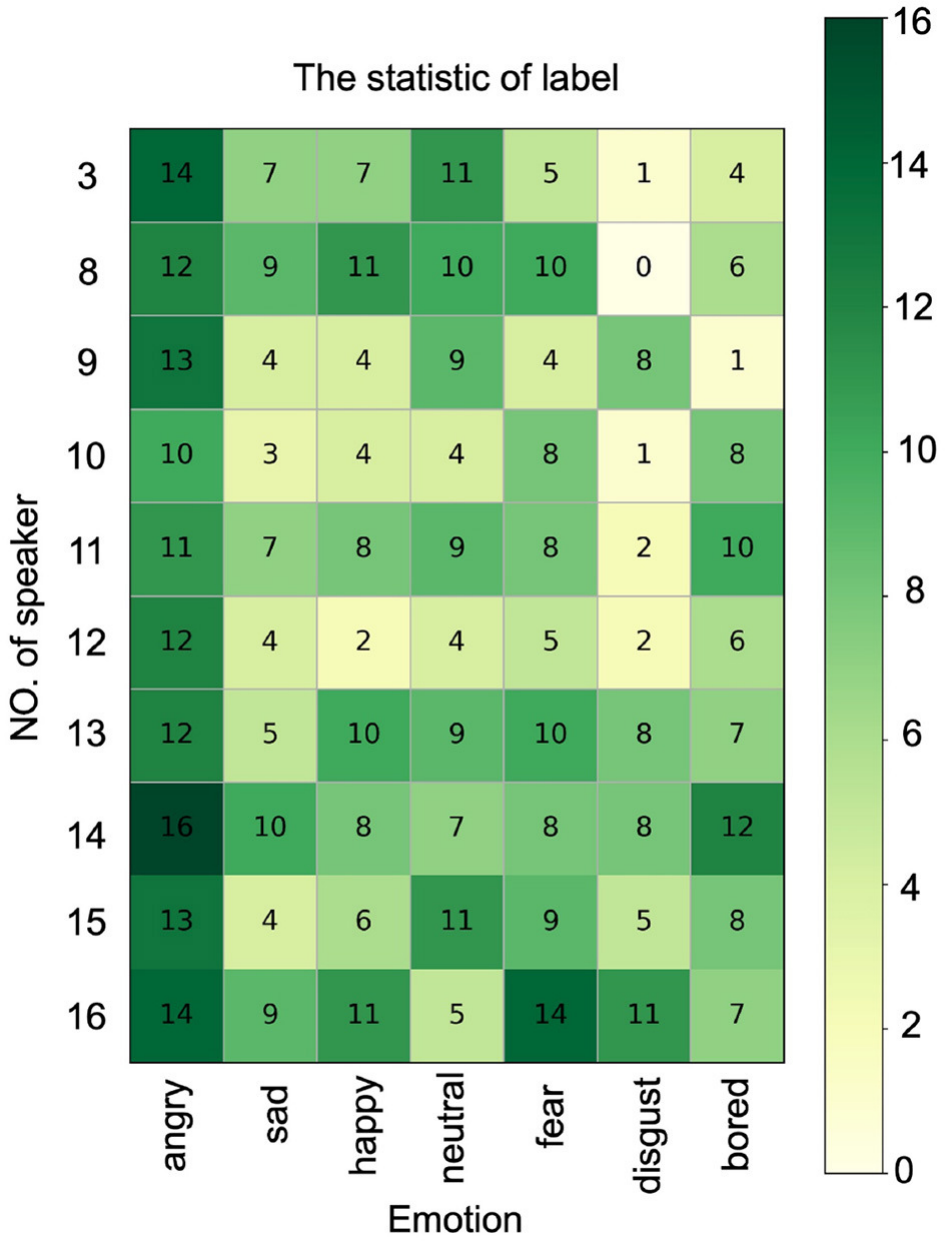
The label statistics of every speaker. The rows represent different speaker. The columns represent different emotions.

SER is a classification task. To evaluate the performance of ResCNN-SER, we perform the speaker-independent experiment on the Emo-DB dataset. We use ten-fold cross-validation to evaluate the proposed model. The Emo-DB dataset contains seven types of emotions, which are considered as seven labels. Besides, the amounts of samples with different labels are different in this dataset. The label statistics are shown in Figure 2. To overcome the bias resulting from unbalanced labels, we use unweighted average recall (UAR) as the model evaluation criteria (Chen et al., 2018). The UAR can be calculated as follows:

(12) $$\begin{array}{r} Recall=\frac{TP}{TP+FN} \end{array}$$

where TP (true positive) represents the number of samples that true label and predicted label are both positive, FN (false negative) represents the number of samples that true label is positive but predicted label is negative. As a complement, we also use accuracy (ACC) as another evaluation metric.

#### 3.1.3. Performance Evaluation of ResCNN-SER

**Figure 3:**
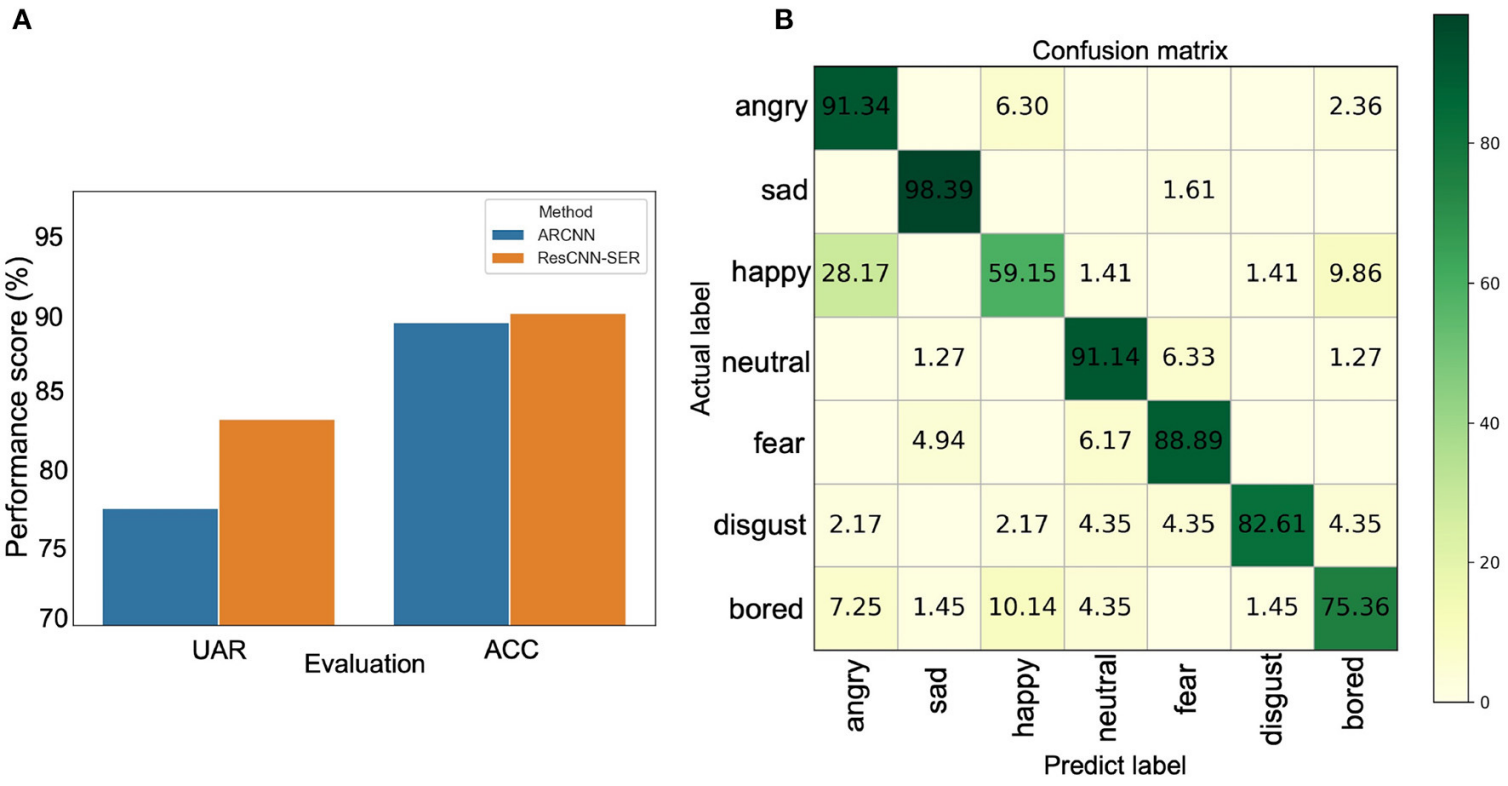
**(A)** The UAR and ACC results of ARCNN and the proposed method ResCNN-SER. The y-axis represents the percentage values of UAR and ACC. The blue bar represents ARCNN method, and the orange bar represents the ResCNN-SER method. **(B)** The confusion matrix of the ResCNN-SER model. The row represents the true label. The column represents the predicted label. The values in the matrix represent percentages of predicted labels in different categories. For example, for the first row, it represents that 91.34, 6.3, and 2.36% of samples labeled as angry are predicted as angry, happy, and bored, respectively.

In this section, we apply the ResCNN-SER model to the Emo-DB dataset to evaluate its performance. We compare the proposed model with the existing state-of-art method, named ARCNN (Chen et al., 2018). The Emo-DB dataset is separated into training set, validation set, and testing set. Comparing with ARCNN, the results show that the performance of ResCNN-SER is consistently better on the Emo-DB dataset using UAR and ACC as evaluation metrics (Figure 3A). The UAR of ResCNN-SER is 0.833, which is about 0.06 higher than ARCNN. Similarly, the ACC of ResCNN-SER is also higher than ARCNN.

Furthermore, the confusion matrix of ResCNN-SER is shown in Figure 3B. It is shown that ResCNN-SER performs well for recognizing most types of emotions. The top four emotions on which ResCNN-SER performs best are sad, angry, neutral, and fear. In summary, ResCNN-SER performs better than the existing state-of-art method.

#### 3.1.4. The Effect of Different Numbers of the Residual Block in ResCNN Model

The residual block is used in the ResCNN-SER model. In this section, we evaluate the effect of different numbers of residual blocks in the ResCNN-SER model. Table 1 shows that ResCNN-SER performs the best if three residual blocks are used in the ResCNN-SER model on the task of predicting speech emotion. Besides, Table 1 indicates that the performance of UAR increases along with the increase of the number of residual blocks. It indicates that the residual block structure can decrease the information loss of the feature extraction process in the ResCNN-SER model.

**Table 1:** The performance of ResCNN model with different numbers of residual blocks.

**Method**	**UAR**
ResCNN with one residual block	0.804
ResCNN with two residual blocks	0.813
ResCNN with three residual blocks	0.833

#### 3.1.5. Effect of Log-Mels Spectrogram Features on the Performance of ResCNN-SER

The log-Mels spectrogram is designed to extract the features of the sound signal. In this study, we use the log-Mels spectrogram of static, deltas, and delta-deltas. To evaluate whether these features can improve the performance of ResCNN-SER, we design an ablation experiment. First, we use static, deltas, or delta-deltas log-Mels spectrogram features as the input of the ResCNN-SER model, respectively. Second, we use the different combinations of two features as the input. Third, we use all three features as the input of the ResCNN-SER model.

Table 2 shows that ResCNN-SER can achieve the best performance (*UAR* = 0.833) if we use all three features. If we use one feature only, the UARs are 0.746, 0.774, and 0.690 for static, deltas, or delta-deltas, respectively. If we use two features, the UARs are 0.805, 0.748, and 0.790 for static and deltas, static and delta-deltas, and deltas and delta-deltas, respectively. In summary, using log-Mels spectrogram features can improve the performance of ResCNN-SER.

**Table 2:** The results of using different combinations of log-Mels spectrogram features.

**Method**	**UAR**
ResCNN with static feature	0.746
ResCNN with deltas feature	0.774
ResCNN with delta-deltas feature	0.690
ResCNN with static and deltas features	0.805
ResCNN with static and delta-deltas features	0.748
ResCNN with deltas and delta-deltas features	0.790
ResCNN with static, deltas, and delta-deltas features	0.833

### 3.2. Association Between Alcohol Addiction and People's Emotion at Court

Using the proposed model ResCNN-SER, we can recognize people's emotions at court. Based on the alcohol addiction history and sounds based emotion of 54 trial records at court, we analyze the association between alcohol addiction and people's emotions at court. In this dataset, people with alcohol addiction problem are all defendants. We analyze the association between alcohol addiction and the defendant's emotion at court. Two datasets of trial records are generated. One includes 27 defendants with alcohol addiction problem. The other includes 27 defendants without alcohol addiction problem. We first apply ResCNN-SER to recognize the defendants' emotions. Then, we analyze the association between alcohol addiction and defendants' emotions.

**Figure 4:**
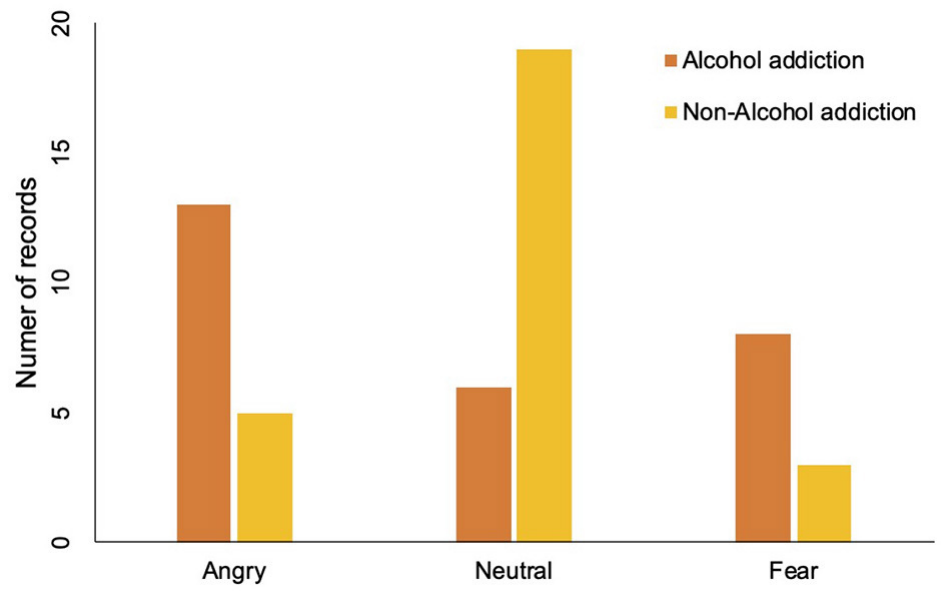
Comparison of alcohol addiction and non-alcohol addiction people on different emotions. The orange bar represents the number of people with alcohol addiction problem. The yellow bar represents the number of people without alcohol addiction problem.

Three types of emotions are recognized from the defendants' voices, which are angry, neutral, and fear. At the court, defendants may express anger because they may not agree that they did something breaking the law. Defendants may express fear because they are afraid of the punishment or worry about their lives are broken. Considering the data availability, we study the association between alcohol addiction and these three types of emotions. The association between alcohol addiction and people's emotions at court is shown in Table 3 and Figure 4. The results show that people with alcohol addiction are more likely to be angry or fearful at court. To be specific, 13 of 27 defendants from the alcohol addiction group show angry emotions at court compared to only five of 27 defendants from the non-alcohol addiction group do. Similarly, eight of 27 defendants show fear emotions in the alcohol addiction group, while only four of 27 defendants show fear emotions in the non-alcohol addiction group. For the neutral emotion, 18 defendants are neutral in the non-alcohol addiction group, while only six of 27 defendants are neutral in the alcohol addiction group. In summary, alcohol addiction can affect the defendants' emotions at the court.

**Table 3:** The association of alcohol addiction and defendant's emotion.

	**Defendant angry**	**Defendant neutral**	**Defendant fear**
Alcohol addiction	13	6	8
Non-alcohol addiction	5	18	4

*The columns represent the defendants' emotions. The rows represent the different data groups, including alcohol addiction group and non-alcohol addiction group. The numbers represent the number of records in the conditions*.

## 4. Conclusion

Recently, AI techniques have been used for solving court matters. For example, an AI-based method is proposed to predict the charges based on the fact descriptions (Hu et al., 2018). This method can be also used in legal assistant systems. In this study, we apply the AI technique to analyze the association between alcohol addiction and people's emotions at court. We propose a novel speech emotions recognition algorithm, named as ResCNN-SER, for predicting the emotions of people at court based on sound perception. The proposed model extracts the features of the speech signals and predicts the emotions by combining several neural network-based components. We first compute the log-Mels spectrogram (static, deltas, and delta-deltas) by taking the speech signals as input. Then, based on the log-Mels spectrogram features, we use a CNN-based model and a long short-term memory-based model to extract the features of speech signals. Next, we apply an attention-based model to enhance the feature representation. Finally, a fully connected neural network layer is used to predict the speech emotion categories. Overall, all evaluation tests show that ResCNN-SER performs better than some existing methods on SER. By using ResCNN-SER for emotion recognition based on sound at court, we analyze the association between alcohol addiction and people's emotions at court. The results show that alcohol addiction can affect the defendants' emotions at court. People with alcohol addiction are more likely to be angry or fearful at court. There are several potential benefits of knowing the association between alcohol addiction and the defendant's emotion. For example, if we know a defendant having an alcohol addiction problem, the judge can ask questions in a more modest way to get more factual statements. We can also make people at the court avoid injury from defendants who easily get angry. Furthermore, we can use cases with alcohol addiction problems as publicity materials to help publicize the harm of alcohol addiction to physical and mental health and the serious consequences of criminal acts such as drunk driving. In the future, we can also use ResCNN-SER to infer the different roles' emotions at court and understand whether the emotions in the voice can affect the defendant. There are still limitations in the application of ResCNN-SER. For example, several people are speaking at the same time at the court and the voice may be recorded on a noisy occasion. In the future, we will develop methods to extract the voice we are interested in from a complex environment at the court.

## Data Availability Statement

The raw data supporting the conclusions of this article will be made available by the authors, without undue reservation.

## Author Contributions

YS designed the study. ZW contributed to design, and with YS carried out the analysis. All authors contributed to the article and approved the submitted version.

## Conflict of Interest

The authors declare that the research was conducted in the absence of any commercial or financial relationships that could be construed as a potential conflict of interest.
